# Reassessing the Link: Depression and Diabetic Nephropathy in Type 2 Diabetes Mellitus Patients: Insights From the ACCORD-HRQL Study

**DOI:** 10.1155/da/1885956

**Published:** 2025-05-05

**Authors:** Dingwu Yi, Zhenhua Xing

**Affiliations:** ^1^Department of Cardiac Surgery, Extracorporeal Life Support Center of Cardiovascular Surgery, Second Xiangya Hospital, Central South University, Changsha, China; ^2^Department of Emergency Medicine, Second Xiangya Hospital, Central South University, Changsha 410011, China; ^3^Emergency Medicine and Difficult Diseases Institute, Central South University, Changsha 410011, China

**Keywords:** depression, diabetic nephropathy, estimated glomerular filtration rate, type 2 diabetes mellitus

## Abstract

**Background:** Type 2 diabetes mellitus (T2DM) patients with depression are often accompanied by diabetic nephropathy. However, limited prospective studies have investigated the independent association between depression and diabetic nephropathy, as well as its progression among T2DM patients. This study aims to investigate the association between depression and the development or progression of diabetic nephropathy in T2DM patients, utilizing data from the ACCORD Health-Related Quality of Life (HRQL) study.

**Methods and Results:** The nine-item Patient Health Questionnaire (PHQ-9) was utilized to assess depressive symptoms at baseline, and at 1, 3, and 4 years. The primary outcomes included deterioration of renal function, macroalbuminuria, and microalbuminuria. The changes in renal function were evaluated using estimated glomerular filtration rate (eGFR). As the severity of depression, measured by the PHQ-9, increased, there was no corresponding rise in the risk of deterioration in renal function (HR, 1.00; 95% CI, 0.98–1.01), macroalbuminuria (HR, 0.99; 95% CI, 0.53–1.86), or microalbuminuria (HR, 1.00; 95% CI, 0.97–1.03) per unit increase in PHQ-9 score. The estimated unadjusted eGFR (mL/min/1.73 m^2^) decline over the entire study period did not significantly differ for each 1-year increase in age (none: 2.21, 95% CI 1.98–2.44; ever depression: 2.51, 95% CI 2.36–2.67; persist depression: 2.28, 95% CI 1.99–2.57; all pairwise *p*-values > 0.05).

**Conclusions:** T2DM patients with depression do not demonstrate lower renal function or an increased rate of renal function decline. Moreover, they do not exhibit a heightened risk of renal function deterioration, macroalbuminuria, or microalbuminuria compared to T2DM patients without depression.

## 1. Background

Patients with type 2 diabetes mellitus (T2DM) face a higher risk of depression compared to their nondiabetic counterparts. The prevalence of depression among patients with T2DM ranges from 20% to 30%, which is nearly twice that of the general population [1, 2]. Depression in T2DM patients is not merely a psychological comorbidity and it directly impacts self-care behaviors, including dietary adherence, physical activity, and medication compliance, leading to poor glycemic control and an increased risk of diabetes-related complications [3–5]. Mechanistically, the bidirectional relationship between T2DM and depression is thought to involve shared pathophysiological pathways, such as chronic inflammation and altered neurotransmitter systems [6].

Moreover, in individuals with T2DM, diabetic nephropathy emerges as the foremost instigator of renal insufficiency and stands as an autonomous contributor to the elevated susceptibility to premature mortality. It affects approximately 20%–40% of individuals with T2DM and is characterized by progressive albuminuria, declining glomerular filtration rate (GFR), and an increased risk of end-stage renal disease (ESRD) [7]. Beyond its impact on renal function, diabetic nephropathy independently contributes to heightened cardiovascular risk and premature mortality, making it a critical concern in T2DM management [8]. Previous studies have highlighted the bidirectional relationship between depression and diabetic nephropathy in individuals with T2DM [9–11]. Depression may exacerbate diabetic nephropathy through pathways, such as chronic inflammation and increased oxidative stress, all of which contribute to endothelial dysfunction and renal injury [12]. Conversely, diabetic nephropathy, characterized by progressive albuminuria and renal insufficiency, can intensify depressive symptoms through mechanisms like chronic pain, fatigue, and the psychological burden of disease management [13]. Most studies, predominantly cross-sectional, and even among the limited prospective research, have focused on the association between depression and ESRD, overlooking the relationship between depression and early stage proteinuria in diabetic nephropathy, as well as the progression of the disease. Specifically, they fail to ascertain whether depression independently acts as a independent risk factor for diabetic nephropathy and its impact on disease progression, quantified by objective laboratory indicators like estimated GFR (eGFR). This is critical given the bidirectional relationship between diabetic nephropathy, especially ESRD and depression [14]. Additionally, depression is a highly dynamic condition, with some patients experiencing recurrent episodes while others show significant improvement or even complete remission. Conversely, individuals without baseline depression may develop moderate-to-severe symptoms over time due to factors, such as chronic disease burden, metabolic changes, or psychosocial stressors. Our previous research highlighted the dynamic nature of depressive symptoms, a factor overlooked in prior studies which did not consistently monitor symptoms during follow-up, indicating a significant research gap [4]. Moreover, the suboptimal blood glucose management and medication adherence observed in T2DM patients with depression represent crucial confounding factors in understanding the depression–diabetic nephropathy association, further compounded by the lack of continuous monitoring in previous investigations, signifying an urgent need for comprehensive longitudinal studies [11].

In light of these previous limitations, our study aims to investigate the association between dynamic depressive symptoms and diabetic nephropathy in individuals with T2DM. Specifically, we will evaluate the impact of depression on key indicators of diabetic nephropathy, including micro- and macroalbuminuria and renal function deterioration. Furthermore, our study accounts for time-varying confounding factors, such as glycated hemoglobin (HbA1c) levels and medication usage, to provide a more nuanced understanding of this relationship. By addressing these gaps, we aim to determine whether depression acts as an independent risk factor for the development and progression of diabetic nephropathy, thereby overcoming the methodological shortcomings of prior research.

## 2. Methods

### 2.1. Study Design

The ACCORD investigation, which recruited 10,251 subjects (with a mean HbA1c of 8.3%) and an average age of 62, aimed to determine whether enhanced management of glucose, blood pressure, and cholesterol could improve cardiovascular health in individuals with T2DM [15]. This study was halted early after a median monitoring period of 3.7 years due to a rise in cardiac death associated with aggressive glucose control [16]. As a result, all participants transitioned to standard glucose management, with monitoring proceeding as originally intended. The ACCORD trial received authorization from the NHLBI review board and the ethics committees of all involved institutions. Importantly, the study's subjects did not contribute to formulating research queries, defining outcome metrics, or developing the study protocol.

Within the ACCORD study, a substudy called the ACCORD Health-Related Quality of Life (HRQL) study sought to prospectively evaluate the effect of interventions on established HRQL metrics from the perspectives of the subjects [17]. Measurements were taken at baseline and at 1, 3, and 4 years. The mean follow-up time was 4.67 ± 1.45 years [17]. Depression symptoms were measured utilizing the nine-item Patient Health Questionnaire (PHQ-9) in accordance with DSM-IV criteria at the commencement and then at 1, 3, and 4 years during clinical assessments within the ACCORD framework. The severity of depression symptoms was classified as none (0–4 points), mild (5–9 points), or moderate-severe (10–24 points) according to PHQ-9 scores [4]. We also categorized the participants into three groups based on their depressive symptom measurements at four time points during the follow-up period: (a) never depressed (never), participants who did not meet the criteria for depression during follow-up period; (b) ever depressed (ever), participants who met the criteria for depression at one or more time points but not consistently; (c) persistently depressed (persist), participants who met the criteria for depression at all measurements, indicating persistent depressive symptoms throughout the follow-up period. Additionally, HbA1c, fasting plasma glucose (FPG), serum creatinine, low density lipoprotein (LDL), high density lipoprotein (HDL), systolic blood pressure (SBP), and, diastolic blood pressure (DBP) were measured every 4 months. In this study, changes in renal function were evaluated using eGFR, which was calculated using the MDRD equation expressed in mL/min/1.73 m^2^. Medication usage for glucose and depression was assessed through annual interviews. The PHQ-9 scores, HbA1c levels, FPG levels, eGFR values, LDL, HDL, SBP, DBP, and medications for lowering glucose and antidepressants were treated as time-varying variables.

### 2.2. Outcomes of Kidney Function

The outcomes of kidney function in the present study included: (1) deterioration of renal function, defined as either a doubling of baseline serum creatinine or a decrease in eGFR of more than 20 mL/min per 1.73 m^2^; (2) macroalbuminuria, defined as a urine albumin/creatinine ratio of ≥33.9 mg/mmol; (3) microalbuminuria, defined as a urine albumin/creatinine ratio of ≥3.4 mg/mmol.

### 2.3. Confounding Factors

**Figure 1:**
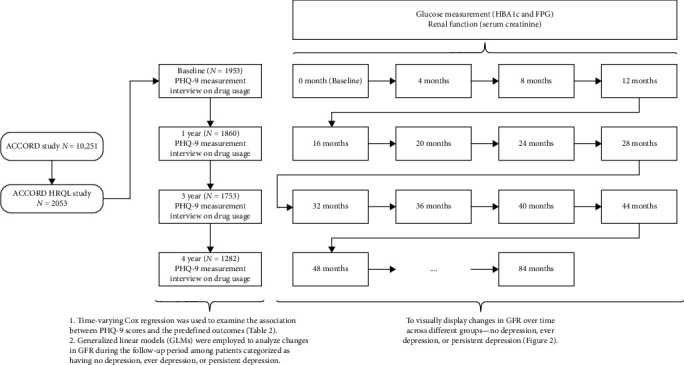
The schematic diagram of the current study.

Baseline participant characteristics, including demographic data, lifestyle habits, and medical histories, were collected via questionnaires, interviews, and medical record examinations during recruitment. These characteristics covered age, gender, ethnicity, T2DM duration, history of cardiovascular disease (CVD) or heart failure, living situations, educational attainment, tobacco and alcohol use, and medication practices. Smoking status was differentiated as never, former, or current smoker, while alcohol usage was self-reported and calibrated by frequency per week. Physiological measures like BMI, SBP, and DBP were measured by certified nurses, and serum creatinine, HbA1c, FPG, and lipid levels were evaluated by the central lab [17, 18]. We utilized two models to investigate the correlations between depression, changes in eGFR, and our defined outcomes. The first model, Model 1, was adjusted for factors, such as age, race, sex, glucose control strategy, FPG, and Hb1Ac. Model 2, on the other hand, incorporated all the elements of Model 1 as well as duration of T2DM, education level, history of cardiovascular disease, heart failure, living conditions, smoking and alcohol consumption, BMI, LDL, HDL, SBP, and DBP. The layout of the present study is illustrated in Figure 1.

### 2.4. Statistical Analysis

Chi-square tests were employed to examine categorical variables, whereas continuous variables were analyzed through either variance analysis or Mann–Whitney *U*-tests based on the nature of their distribution. The Generalized Linear Mixed-Effects Model (GLMM) is an enhanced version Generalized Linear Model (GLM) by integrating random effects with the usual fixed effects in the linear predictor. This model excels in its capacity to analyze outcomes that are measured multiple times over time for the same subjects. Moreover, it is capable of examining grouped data across time by treating the differences between groups (none, ever, persist) as random effects [19]. We analyzed the variance in eGFR decline among the groups adjusted for Model 2 during the follow-up periods. Time-varying Cox proportional hazards regression with PHQ-9 (both as a categorical and continuous variable) was used to assess the association between depression and our predefined outcomes using the two models mentioned above. In addition to Model 2, we included medications as time-dependent covariates to test whether glucose-lowering agents and antidepressants affect the association between depression and our predefined outcomes. The subgroup and interaction analyses were performed according to age (≤60 years, >60 years), sex, glycemic control strategy (intensive or standard glucose control), and CVD history. We conducted a further sensitivity analysis, including patients with complete PHQ-9 assessments from all four evaluations, to examine the potential impact of data loss on the analysis. All statistical analyses were two-sided, with *p*  < 0.05 considered statistically significant. Analyses were performed using Stata/MP version 17.0 (StataCorp LLC, College Station, TX, USA).

## 3. Results

Among the 10,251 participants in the ACCORD study, 2053 were included in the ACCORD-HRQL study and subsequently in our analysis. Among them, baseline PHQ-9 measurements were obtained for 1953 participants (95%). At the 1-year mark, 1860 participants (91%) completed the measurements, while at the 3-year mark, 1753 participants (85%) did so. By the 4-year mark, 1282 participants (62%) had completed the measurements. Over the 4-year follow-up, depression developed in 1267 participants, with 439 participants experiencing persistent depression. Participants with depression tended to be younger, female, obese, have a history of CVD or heart failure, smoke, exhibit higher levels of HbA1c, FPG, LDL, eGFR, lower levels of HDL, and receive antidepressant treatment (Figure S1) compared to those without depression (Table 1).

**Figure 2:**
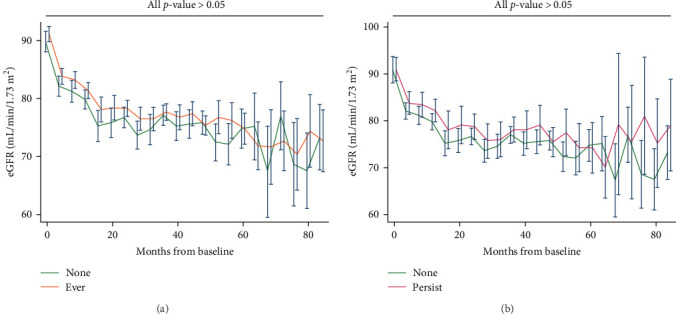
Changes in eGFR and corresponding 95% confidence intervals during the follow-up period in participants with type 2 diabetes mellitus (T2DM) across different depression statuses: (a) Participants without depression (none) vs. participants with a history of depression (ever), and (b) Participants without depression (none) vs. participants with persistent depression (persist).

Participants who experienced depression did not exhibit lower eGFR compared to their counterparts who did not (Figure 2A); similarly, patients with persistent depression did not show lower eGFR compared to their counterparts without depression (Figure 2B). The estimated unadjusted eGFR decline over the entire study period did not significantly differ for each 1-year increase in age (None: 2.21, 95% CI, 1.98–2.44; Ever: 2.51, 95% CI, 2.36–2.67; Persist: 2.28, 95% CI, 1.99–2.57; all pairwise *p*-values > 0.05). The estimated adjusted eGFR (Model 2) showed similar results (none: 2.48, 95% CI, 2.15–2.82; ever: 2.79, 95% CI 2.58–3.00; persist: 2.45, 95% CI 2.05–2.85; all pairwise *p*-values > 0.05).

We further evaluated the association between depression and our predefined kidney outcomes after adjusting for confounding factors. Compared with participants without depression, those with mild or moderate-severe depression did not show a higher risk of deterioration in renal function, macroalbuminuria, or microalbuminuria (Table 2). Interestingly, with increasing severity of depression, there was no corresponding increase in the risk of deterioration in renal function (HR, 1.00; 95% CI, 0.98–1.01), macroalbuminuria (HR, 0.99; 95% CI, 0.53–1.86), or microalbuminuria (HR, 1.00; 95% CI, 0.97–1.03) per unit increase in PHQ-9 score. After further adjusting for glucose-lowering medications and antidepressants, we found similar results: depression, as evaluated by the PHQ-9, was not associated with a higher risk of deterioration in renal function, macroalbuminuria, or microalbuminuria (Table 2). In the subgroup analysis, we did not find age, sex, race, glucose-lowering strategy, or CVD history to exert an interactive effect between depression and our predefined outcomes (Table S1). We conducted a further sensitivity analysis, including patients with complete PHQ-9 assessments from all four evaluations, and found that depression, as assessed by the PHQ-9, was not associated with an increased risk of renal function deterioration, macroalbuminuria, or microalbuminuria (Table S2).

## 4. Discussion

In this new analysis of data from the ACCORD-HRQL trial, we uncovered significant findings among participants with type 2 diabetes: (1) patients with T2DM and depression did not show lower eGFR or a higher rate of eGFR decline compared to their counterparts without depression; (2) upon adjustment for relevant confounding factors, T2DM patients with depression did not exhibit a higher risk of renal function deterioration, macroalbuminuria, or microalbuminuria.

Our study provides evidence that among individuals with T2DM, depression is not independently associated with lower eGFR, faster eGFR decline, or a higher risk of renal function deterioration, macroalbuminuria, or microalbuminuria. These findings suggest that depression may not directly influence the progression of diabetic nephropathy, contrasting with the widely held belief derived from cross-sectional studies. Most cross-sectional studies have found an association between depression and diabetic nephropathy, despite some controversial results [6, 7]. However these studies might have overestimated the impact of depression on renal function by failing to determine whether depression preceded diabetic nephropathy or if diabetic nephropathy contributed to the subsequent onset of depression. A key strength of our study is the precise determination of both diabetic nephropathy status and depression status at fixed time intervals throughout the study period. Additionally, the poor blood glucose management and medication adherence seen in T2DM patients with depression are significant confounding factors in the depression–diabetic nephropathy relationship. Several factors may explain why depression was not associated with renal outcomes in our study. First, intensive clinical management in the ACCORD-HRQL trial may have mitigated the potential impact of depression on renal function. Second, it is possible that previous associations observed in cross-sectional studies were confounded by poor glycemic control, medication adherence, or other unmeasured factors. Lastly, depression might influence health outcomes indirectly rather than directly affecting renal function, necessitating further investigation into behavioral and psychosocial mechanisms.

Compared to previous studies, we effectively accounted for a wide range of baseline and follow-up clinical characteristics associated with diabetic nephropathy, including time-varying HbA1c, FPG, SBP, DBP, medications for lowering glucose and antidepressants, as well as various social and behavioral factors that could potentially confound the relationship between depression and the studied outcomes, such as smoking, alcohol consumption, living arrangements, and educational level [20]. In clinical settings, depressive symptoms change dynamically [4]. However, most studies only examine the cross-sectional correlation, and even the few prospective cohort studies [10] have not reevaluated these symptoms to consider such dynamic changes. In contrast, our study conducted four repeated assessments, thereby providing a more accurate evaluation of patient symptoms. The most important aspect is that our study observed in real time the impact of depression and its severity on changes in kidney function. We did not find any influence of depression on change in kidney function, thus corroborating our conclusion that depression does not affect diabetic nephropathy. Our findings emphasize the importance of addressing depression in diabetes care, not necessarily for its direct impact on renal function but for its broader effects on quality of life and overall health. Future research should focus on elucidating the indirect pathways through which depression affects health outcomes and on identifying subgroups of patients who may be more vulnerable to its effects.

We study have several limitations. Firstly, it is a post hoc analysis of an RCT. Consequently, the management of HbA1c was strictly controlled by the researchers, which may not fully reflect real-world conditions. In other words, in the real world, T2DM patients with depression may exhibit worse HbA1c compared to counterparts in the present study. Secondly, a significant number of participants were lost to follow-up for the PHQ-9 data in the fourth year. Nevertheless, we found no significant differences in baseline characteristics between the lost-to-follow-up patients and those with complete data. Additionally, our present study might better reflect patients' depressive symptoms compared to previous studies that only had baseline PHQ-9 data. Lastly, it's worth noting that all participants in our study were recruited from the United States and Canada. Therefore, the generalizability of our findings to other populations may be limited.

## 5. Conclusions

In conclusion, individuals with T2DM who also experience depression do not demonstrate lower renal function or an increased rate of renal function decline. Moreover, they do not exhibit a heightened risk of renal function deterioration, macroalbuminuria, or microalbuminuria compared to T2DM patients without depression.

## Figures and Tables

**Table 1:** Baseline characteristics of participants based on depression status.

Depression status	All	Ever	Persist
*N*	1953	1267	439
Age (years)	62.80 ± 6.60	62.30 ± 6.70*⁣*^*∗*^	61.62 ± 6.58*⁣*^*∗*^
Sex, female (%)	778 (39.84%)	555 (43.80%)*⁣*^*∗*^	206 (46.92%)*⁣*^*∗*^
Race, White (%)	1248 (63.90%)	822 (64.88%)	295 (67.20%)*⁣*^*∗*^
CVD history (%)	701 (35.89%)	493 (38.91%)*⁣*^*∗*^	173 (39.41%)*⁣*^*∗*^
Living alone (%)	392 (20.07%)	273 (21.55%)	104 (23.69%)*⁣*^*∗*^
Education (%)	—	*⁣* ^*∗*^	*⁣* ^*∗*^
Less than high school	273 (13.99%)	208 (16.43%)	64 (14.58%)
High school graduate	509 (26.09%)	324 (25.59%)	114 (25.97%)
Some college	640 (32.80%)	423 (33.41%)	146 (33.26%)
College degree or higher	529 (27.11%)	311 (24.57%)	115 (26.20%)
Duration of T2DM (years)	11.03 ± 7.70	11.36 ± 7.96*⁣*^*∗*^	11.32 ± 8.08
Heart failure (%)	100 (5.12%)	76 (6.00%)*⁣*^*∗*^	32 (7.29%)*⁣*^*∗*^
Current Smoker (%)	255 (13.06%)	185 (14.60%)*⁣*^*∗*^	72 (16.40%)*⁣*^*∗*^
Alcohol consumption per week	0.93 ± 2.61	0.80 ± 2.40*⁣*^*∗*^	0.74 ± 2.25*⁣*^*∗*^
BMI (Kg/m^2^)	32.43 ± 5.39	33.00 ± 5.50*⁣*^*∗*^	33.97 ± 5.53*⁣*^*∗*^
FPG (mg/dL)	176.65 ± 57.5	179.90 ± 59.03*⁣*^*∗*^	182.39 ± 60.15*⁣*^*∗*^
HbA1c (%)	8.28 ± 1.05	8.35 ± 1.06*⁣*^*∗*^	8.42 ± 1.09*⁣*^*∗*^
Blood pressure	—	—	—
SBP (mmHg)	136.30 ± 17.05	136.18 ± 16.89	135.53 ± 16.45
DBP (mmHg)	74.43 ± 10.90	74.76 ± 11.16*⁣*^*∗*^	74.65 ± 10.89
Lipid	—	—	—
LDL (mg/dL)	104.16 ± 33.75	104.63 ± 34.49*⁣*^*∗*^	105.72 ± 35.57*⁣*^*∗*^
HDL (mg/dL)	42.14 ± 11.60	41.92 ± 11.71*⁣*^*∗*^	41.11 ± 11.25*⁣*^*∗*^
GFR (mL/min/1.73 m^2^)	91.73 ± 31.16	92.16 ± 27.22	91.01 ± 26.50
Medications	—	—	—
TZD (%)	457 (23.28%)	312 (24.63%)	108 (24.60%)
Metformin (%)	1254 (63.88%)	815 (64.33%)	278 (63.33%)
Sulfonylurea (%)	1051 (53.54%)	663 (52.33%)	199 (45.33%)*⁣*^*∗*^
Meglitinide (%)	41 (2.09%)	25 (1.97%)	9 (2.05%)
Insulin (%)	228 (11.61%)	156 (12.31%)	66 (15.03%)*⁣*^*∗*^
ACEI (%)	1041 (53.3%)	675 (53.3%)	249 (56.7%)
ARB (%)	343 (17.6%)	222 (17.5%)	74 (16.8%)
Antidepressants	288 (14.1%)	236 (18.6%)*⁣*^*∗*^	119 (27.1%)*⁣*^*∗*^

*Note:* Continuous data are presented as mean ± SD, and categorical data as *n* (%). Categorical variables were analyzed using *χ*² tests, while continuous variables were compared using either analysis of variance or the Mann–Whitney *U*-test, depending on their distribution.

Abbreviations: ACEI, angiotensin converting enzyme inhibitor; ARB, angiotensin receptor blockers; BMI, body mass index; CVD, cardiovascular disease; DBP, diastolic blood pressure; FPG, fasting plasma glucose; GFR, glomerular filtration rate; HbA1c, glycosylated hemoglobin A1c; HDL, high density lipoprotein; LDL, low density lipoprotein; SBP, systolic blood pressure; T2DM, type 2 diabetes mellitus; TZD, thiazolidinediones.

*⁣* ^*∗*^Indicates statistical significance compared to participants without depression.

**Table 2:** Association between depression and predefined renal outcomes in T2DM.

Study outcomes	Depression groups	Model 1	Model 2	Model 2 + medications
Deterioration in renal function	None	Ref	Ref	Ref
	Mild	0.97 (0.83,1.12)	0.95 (0.82,1.10)	0.96 (0.83,1.12)
	Moderate–severe	1.05 (0.88,1.24)	1.01 (0.85,1.21)	1.02 (0.85,1.23)
	*p* for trend	0.75	0.83	0.95
	PHQ-9 continuous	1.00 (0.99,1.01)	1.00 (0.98,1.01)	1.00 (0.98,1.01)

Macroalbuminuria	None	Ref	Ref	Ref
	Mild	1.21 (0.75,1.93)	1.21 (0.75,1.96)	1.20 (0.74,1.95)
	Moderate–severe	1.15 (0.63,2.09)	0.99 (0.53,1.86)	0.99 (0.52,1.89)
	*p* for trend	0.68	0.81	0.81
	PHQ-9 continuous	1.03 (0.98,1.07)	1.02 (0.97,1.07)	1.02 (0.97,1.07

Microalbuminuria	None	Ref	Ref	Ref
	Mild	1.19 (0.89,1.58)	1.12 (0.83,1.51)	1.11 (0.82,1.50)
	Moderate–severe	1.27 (0.89,1.81)	1.07 (0.74,1.55)	1.07 (0.73,1.57)
	*p* for trend	0.13	0.57	0.60
	PHQ-9 continuous	1.02 (0.99,1.04)	1.00 (0.97,1.03)	1.00 (0.97,1.03)

*Note:* Time-varying Cox proportional hazards regression was used to assess the relationship between depression and the outcomes of kidney function. Model 1 was adjusted for factors, such as age, race, sex, glucose control strategy, FPG, and HbA1c. Model 2, on the other hand, incorporated all the elements of Model 1 in addition to duration of T2DM, education level, history of cardiovascular disease, heart failure, living conditions, smoking and alcohol consumption, BMI, LDL, HDL, SBP, and DBP. Values represent hazard ratios with 95% confidence intervals.

Abbreviations: CVD, cardiovascular disease; DBP, diastolic blood pressure; FPG, fasting plasma glucose; HbA1c, glycosylated hemoglobin A1c; HDL, high density lipoprotein; LDL, low density lipoprotein; PHQ-9, nine-item Patient Health Questionnaire; SBP, systolic blood pressure.

## Data Availability

Data are available from the Biologic Specimen and Data Repository Information Coordinating Center (BioLINCC), https://www.nhlbi.nih.gov/.

## Supporting Information

Supporting Information Figure S1. Percentage of antidepressant use based on depression status. Supp orting Information. Table S1. Subgroup analysis based on age, sex, race, glucose-lowering strategy, and cardiovascular disease (CVD) history. Supp orting Information. Table S2. Association between depression and predefined renal outcomes in patients with complete data from all four measurements.
