# Supplementary material for: Reassessing the Link: Depression and Diabetic Nephropathy in Type 2 Diabetes Mellitus Patients: Insights From the ACCORD-HRQL Study
Source: Depress Anxiety. 2025 May 5;2025:1885956. doi: 10.1155/da/1885956 (PMC12069834; doi:10.1155/da/1885956)

**Supplementary Table 1: Subgroup analysis.**

| **Subgroups** | **Outcomes** |  | **HR and 95%CI per 1 increase in PHQ-9** | **P for interaction** |
| --- | --- | --- | --- | --- |
| Age | Deterioration in renal function | Age>=60 | 1.00(0.98,1.02) | 0.96 |
|  |  | Age<60 | 1.00(0.98,1.02) |  |
|  | Macro-albuminuria | Age>=60 | 0.99(0.92,1.07) | 0.27 |
|  |  | Age<60 | 1.04(0.98,1.10) |  |
|  | Micro-albuminuria | Age>=60 | 1.01(0.97,1.06) | 0.74 |
|  |  | Age<60 | 1.01(0.97,1.04) |  |
| Sex | Deterioration in renal function | Female | 1.00(0.97,1.02) | 0.82 |
|  |  | Male | 1.00(0.98,1.02) |  |
|  | Macro-albuminuria | Female | 1.01(0.93,1.09) | 0.58 |
|  |  | Male | 1.03(0.97,1.08) |  |
|  | Micro-albuminuria | Female | 1.01(0.96,1.06) | 0.65 |
|  |  | Male | 1.00(0.96,1.04) |  |
| Race | Deterioration in renal function | White | 0.99(0.98,1.01) | 0.74 |
|  |  | Non-White | 1.00(0.98,1.02) |  |
|  | Macro-albuminuria | White | 1.01(0.95,1.08) | 0.32 |
|  |  | Non-White | 1.04(0.98,1.11) |  |
|  | Micro-albuminuria | White | 1.00(0.96,1.03) | 0.57 |
|  |  | Non-White | 1.02(0.97,1.07) |  |
| Glucose control strategy | Deterioration in renal function | Intensive | 0.99(0.97,1.01) | 0.65 |
|  |  | Standard | 1.00(0.98,1.02) |  |
|  | Macro-albuminuria | Intensive | 1.03(0.96,1.10) | 0.89 |
|  |  | Standard | 1.01(0.95,1.08) |  |
|  | Micro-albuminuria | Intensive | 1.02(0.98,1.06) | 0.06 |
|  |  | Standard | 0.97(0.93,1.01) |  |
| CVD history | Deterioration in renal function | Yes | 1.01(0.98,1.03) | 0.51 |
|  |  | No | 0.99(0.98,1.01) |  |
|  | Macro-albuminuria | Yes | 1.04(0.97,1.11) | 0.77 |
|  |  | No | 1.01(0.95,1.08) |  |
|  | Micro-albuminuria | Yes | 0.96(0.91,1.00) | 0.06 |
|  |  | No | 1.03( 0.99,1.07) |  |

**Supplementary Table 2: Association between depression and predefined renal outcomes in patients with complete data from all four measurements.**

|  |  | Model 1 | Model 2 |
| --- | --- | --- | --- |
| Deterioration in renal function | None | Ref | Ref |
|  | Mide | 0.97(0.81,1.15) | 0.94(0.78,1.13) |
|  | Moderate-severe | 1.11(0.90,1.36) | 1.11(0.90,1.37) |
|  | P for trend | 0.45 | 0.60 |
|  | PHQ-9 continuous | 1.01(0.99,1.02) | 1.01(0.99,1.02) |
| Macro-albuminuria | None | Ref | Ref |
|  | Mide | 1.00(0.59,1.72) | 0.98(0.57,1.68) |
|  | Moderate-severe | 1.01(0.51,2.01) | 0.91(0.45,1.83) |
|  | P for trend | 0.98 | 0.80 |
|  | PHQ-9 continuous | 1.01(0.96,1.06) | 1.00(0.95,1.05) |
| Micro-albuminuria | None | Ref | Ref |
|  | Mide | 1.19(0.89,1.58) | 1.17(0.84,1.62) |
|  | Moderate-severe | 1.27(0.75,1.83) | 1.31(0.88,1.93) |
|  | P for trend | 0.13 | 0.15 |
|  | PHQ-9 continuous | 1.02(0.99,1.05) | 1.01(0.98,1.04) |

*PHQ-9,nine-item Patient Health Questionnaire; CVD,cardiovascular disease; FPG,fasting plasma glucose; HbA1c,glycosylated hemoglobin A1c; SBP,systolic blood pressure; DBP,diastolic blood pressure; CHOL,total cholesterol; TRIG,Triglyceride; LDL,low density lipoprotein; HDL,high density lipoprotein.*

**Supplementary Figure 1: Percentage of antidepressant use based on depression status.**


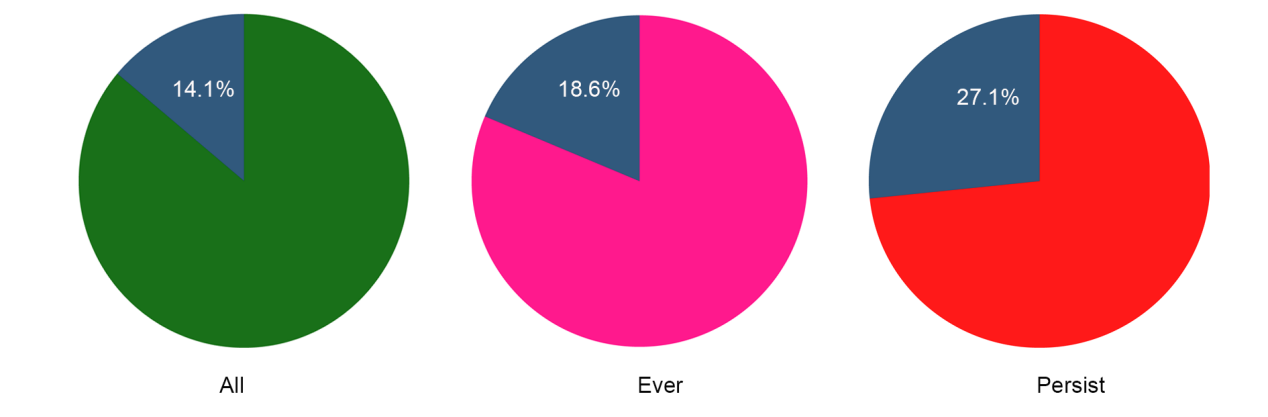

Supplement: Supporting Information — Figure S1. Percentage of antidepressant use based on depression status. Supp orting Information. Table S1. Subgroup analysis based on age, sex, race, glucose-lowering strategy, and cardiovascular disease (CVD) history. Supp orting Information. Table S2. Association between depression and predefined renal outcomes in patients with complete data from all four measurements. [file 1885956.f1.docx]
